# A Case of Vertebral Dislocation with Favorable Termination

**Published:** 1855-04

**Authors:** J. U. Hickerman

**Affiliations:** Tiffin City, Ohio


*A Case of Vertebral Dislocation, with Favorable Termination.*
By J. U. Hickerman, M. D, Tiffin City, Ohio.

On the 11th of December, 1852, as I was returning from a ride to the country, in passing R. Good's residence, I was accosted by his lady with, "Oh! Doctor, come in quick; you were sent for, but being absent we have sent for another Doctor, who, however, has not yet arrived."

Upon entering the house I found a female of considerable more than ordinary size lying in bed upon her back, in an extended position, with the head slightly turned to the right. The surface of the body was but little below the natural temperature, pupils dilated, pulse absent at the wrist and elbow, heart's action feeble and scarcely perceptible, respiration absent, except an occasional (one in about two or two and a half minutes) gasp.

I learned from the bystanders that the patient had been thrown from a wagon, the wheels of which had traversed the epigastric and hypochondriac regions of her body.

At first I looked upon the case as one in which organic life was partially suspended, as a result of the shock produced by the fall; and dispatched a person to a neighboring grocery for some brandy, which arrived simultaneous with Dr. E. Reeme, of this city, who had been summoned during my absence.

The brandy was given, first cautiously, afterwards more liberally, without at all, for some time, effecting the circulation. I now advised Prof. Good and lady to send for the friends of the patient, (she being a domestic in the family.) inasmuch as we feared the existence of internal hæmorrhage, and that death would ensue, without a probability of surgical relief being afforded.

After the lapse of some time the pulse became perceptible at the wrist, at first feeble, but soon distinct and of moderate fullness, but irregular, and in quantity of from sixty to ninety to the minute, not beating at the same rate for more than from five to fifteen continuous seconds.

I now suggested to Dr. R. the propriety of artificial respiration, which was commenced and continued for about the space of half an hour.

Vertebral dislocation, at this stage of our proceedings, suggested itself to my mind, and thrusting a finger into the pharnyx of the patient, discovered a well marked, and easily distinguished prominence, at the junction of the fourth and fifth vertebra.

Upon this discovery I said to Dr. R: "If you put your finger down her throat you may detect the nature of the case." Upon examination Dr. R. said: "it (the protuberance) was very well marked."

After a little reflection I seized the patient's head under my left arm, with which I made traction, and with the index finger of the right hand in the patient's throat, I made firm pressure obliquely upward, backward and to the left; after continuing this pressure for about forty to fifty seconds, the part against which the finger was placed gradually, yet quickly, receded in the direction in which the pressure was made, and instantly as quickly indeed as the act could possiply be executed, the patient opened her eyes, and natural respiration was established. The patient immediately became conscious of what was transpiring about her, recognized the persons present, and upon being asked if she was suffering any pain, signified by signs (being unable to speak.) that she had, and when asked its locality, referred to the epigastric and hypochondriac regions.

Two hours after the above occurrence I again saw the patient. and found her comparatively comfortable, with a pulse of 80, somewhat small and hard, and respiration nearly natural. At 7 o'clock P. M., eight hours after the occurrence of the accident, reaction had ensued, accompanied with hot surface, accelerated breathing quick, full pulse and thirst; blood was now abstracted to the amount of about 25 oz, a saline cathartic or'eied. and Tart. Emet given in solution in sedative doses. and the patient was speedily restored to perfect health, which has been uninterruptedly enjoyed up to the present time, a period of twenty-six months.—*Ohio Med. and Surg. Journal.*

